# Characterization of T cell receptor repertoire in penile cancer

**DOI:** 10.1007/s00262-023-03615-z

**Published:** 2024-01-27

**Authors:** Junying Zhang, Yapeng Wang, Yiqiang Huang, Xintao Tan, Jing Xu, Qian Yan, Jiao Tan, Yao Zhang, Jun Zhang, Qiang Ma, Hailin Zhu, Jin Ye, Zhaojing Zhu, Weihua Lan

**Affiliations:** 1 Chongqing Key Laboratory of High Active Traditional Chinese Drug Delivery System, Chongqing Engineering Research Center of Pharmaceutical Sciences, Chongqing Medical and Pharmaceutical College, Chongqing, 401331 People’s Republic of China; 2 College of Pharmacy, Chongqing Medical University, Chongqing, 400016 People’s Republic of China; 3 Department of Urology, Daping Hospital, Army Medical University, Chongqing, 400042 People’s Republic of China; 4 Urinary Nephropathy Center, The Thirteenth People’s Hospital of Chongqing, Chongqing, 400053 People’s Republic of China; 5 School of Pharmacy, Chongqing Medical and Pharmaceutical College, Chongqing, 401331 People’s Republic of China

**Keywords:** Penile cancer, Tumor microenvironment, High-throughput sequencing, TCR repertoire, TCR clonality

## Abstract

Tumor-infiltrating lymphocytes (TILs) play a key role in regulating the host immune response and shaping tumor microenvironment. It has been previously shown that T cell infiltration in penile tumors was associated with clinical outcomes. However, few studies have reported the T cell receptor (TCR) repertoire in patients with penile cancer. In the present study, we evaluated the TCR repertoires in tumor and adjacent normal tissues from 22 patients with penile squamous cell carcinoma (PSCC). Analysis of the T cell receptor beta-variable (TRBV) and joining (TRBJ) genes usage and analysis of complementarity determining region 3 (CDR3) length distribution did not show significant differences between tumor and matched normal tissues. Moreover, analysis of the median Jaccard index indicated a limited overlap of TCR repertoire between these groups. Compared with normal tissues, a significantly lower diversity and higher clonality of TCR repertoire was observed in tumor samples, which was associated with clinical characteristics. Further analysis of transcriptional profiles demonstrated that tumor samples with high clonality showed increased expression of genes associated with CD8 + T cells. In addition, we analyzed the TCR repertoire of CD4 + T cells and CD8 + T cells isolated from tumor tissues. We identified that expanded clonotypes were predominantly in the CD8 + T cell compartment, which presented with an exhausted phenotype. Overall, we comprehensively compared TCR repertoire between penile tumor and normal tissues and demonstrated the presence of distinct T cell immune microenvironments in patients with PSCC.

**Supplementary Information:**

The online version contains supplementary material available at 10.1007/s00262-023-03615-z.

## Introduction

Penile carcinoma is a rare cancer in high-income countries. However, it may constitute up to 10% of male malignancies in developing countries, including African, Asian, and South American countries [1]. Approximately 95% of penile cancers are penile squamous cell carcinoma (PSCC) [2, 3]. Patients with advanced loco-regional disease or distant metastasis have poor prognosis and high morbidity [3, 4]. Although cisplatin-based chemotherapy offers better outcomes, long-term remission is rare, with overall survival lower than 12 months [5]. Significant response to immunotherapy with atezolizumab has been observed in a group of PSCC patients with high-risk human papillomavirus (HPV) and high infiltration of intratumoral CD3 + CD8 + T cells [6], evidencing the importance of tumor‑infiltrating T lymphocytes in PSCC.

T cells play a significant role in the adaptive immune response and are closely related to the anti-tumor immune response and clinical outcome in distinct types of cancer. Antigen recognition depends on the interaction of T cell receptor (TCR) with its cognate peptide bound to major histocompatibility complex molecules [7]. TCRs are heterodimers, typically consisting of α and β chains. The specificity and diversity of TCRs predominantly depend on the highly variable complementarity determining region 3 (CDR3) and random rearrangement of germline variable (V), diversity (D), and joining (J) segments [8, 9]. Therefore, targeted sequencing of the CDR3 region of the TCRβ chain is useful to identify T cell clonotypes, their frequencies, and the existence of antigenic responses within a repertoire [10]. Studies have characterized the signatures of TCR repertoire in different cancers and confirmed that it could serve as a biomarker for immune response and possess important diagnostic and prognostic value [11–13]. Although some studies have shown that CD8 + T cell infiltration is associated with better clinical prognosis in patients with PSCC [6], the characteristics of the TCR repertoire in patients with PSCC and the relationship between the TCR repertoire and clinical features remain unknown.

In the present study, we performed high-throughput TCR sequencing to characterize TCR repertoires in tumor samples and adjacent normal tissues from patients with PSCC. Our results showed that TCR clonality was associated with tumor malignant behavior, which might be associated with exhausted CD8 + T cells expressing high levels of T cell immunoglobulin mucin receptor 3 (TIM-3) and lymphocyte activation gene 3 (LAG-3). These findings indicated that TCR repertoires could be important biomarkers for clinical prognosis of PSCC and provide a rational basis for further investigation of immune checkpoint blockade (ICB) therapy targeting TIM-3 or LAG-3 in patients with PSCC.

## Materials and methods

### Sample collection

Twenty-two tumor tissue samples and 16 paired adjacent normal tissues from 22 patients with PSCC were obtained from the Daping Hospital of Army Medical University in Chongqing, China. They did not undergo chemotherapy or radiotherapy before sampling and their clinical characteristics are shown in Table 1 and Dataset S1.

**Table 1:** Clinical characteristics of 22 squamous cell carcinoma patients

Parameter	Results (*N* = 22)
Median (range) age, years	67 (33–83)
Histological subtype	
Usual	17 (72.3%)
Other1	5 (22.7%)
Histopathological grade	
Well	12 (54.5%)
Moderately	6 (27.3%)
Pooly	6 (18.2%)
Tumor (T) stage	
T1	8 (36.3%)
T2	7 (31.8%)
T3	7 (31.8%)
HPV status	
Positive	19 (86.4%)
Negative	3 (13.6%)

^1^Other includes basaloid, warty, papillary, and sarcomatoid subtypes

### HPV genotyping

HPV types were detected using the Tellgenplex™ HPV DNA Test (Tellgen Life Science Co. Ltd., Shanghai, China). The assay can be used to qualitatively detect 14 HPV genotypes (including HPV 16, 18, 31, 33, 35, 39, 45, 51, 52, 56, 58, 59, 66, 68) in a single test. Briefly, in each reaction mixture, 100 ng genomic DNA was used as template and added into 20 μl reaction mixture that includes PCR reaction solution, oligonucleotide primers, and fluorescent labeled probes.

### TCR sequencing and data analysis

Total RNA was isolated from 50 to 100 mg of tissue using Trizol reagent (Invitrogen, USA) according to the manufacturer’s instruction. The quality and quantity of RNA were measured by using a NanoDrop spectrophotometer (Thermo Fisher Scientific, USA). Then, a total of 1–10 μg RNA was converted to cDNA by using RevertAid™ First Strand cDNA Synthesis Kit (Thermo) with a universal primer (RT primer: 5′- AACACAGCGACCTCGGGTG-3′). A two-round multiplex PCR approach was used to amplify the CDR3 region of the TCRβ chains. Specifically, a set of forward primers (each specific to one or a set of functional TCR Vβ segments) and a reverse constant region-specific primer (Dataset S2a) was used to the first PCR and generated amplicons covering the entire CDR3 region. The second PCR was performed using a proprietary barcode sequence and Illumina adapter sequences (Dataset S2b). PCR products were identified on 2% agarose gels, and bands between 240 and 350 bp in size were excised and purified using QIAquick Gel Extraction kit (Qiagen, Valencia, CA, USA). Purified products were sequenced using the Illumina Novaseq platform.

Raw sequence data from high-throughput sequencing were stored in FASTQ format. After filtering low-quality sequences, the MiXCR software (version 3.0.13) was used to perform the CDR3 sequence extraction, V, D, and J gene identification, and errors correction. Then, the obtained repertoires were filtered to discard the ambiguous V-beta- and J-beta-segment alignment, out-of-frame and stop codon-containing CDR3 variants. Finally, the resulting sequences were selected for further analysis using the immunarch (version 0.6.5) R package.

The Jaccard index was used to measure the clonotype overlap between samples, regardless of their respective frequency [14]. The Morisita–Horn similarity index was used to determine similarities of TCR repertoires between samples, taking into account the number of shared sequences between two repertoires and the contribution of those shared sequences to each repertoire. To standardize the sample size, each sample was down sampled to the smallest number of sample size. And, we repeated random resampling 100 times using the R program to obtain the median index used to determine the Jaccard and Morisita–Horn similarity indices.

The inverse Simpson diversity index was used to evaluate the TCR diversity. To eliminate the impact of the sample size on the diversity level, the number of TCRβ sequence reads was standardized for each sample down to the smallest number of sample size prior to calculating diversity index. We repeated random resampling 100 times using the R program to obtain the median index used to determine the diversity index for the sample as previously described [15]. The value ranges from 0 to 1, with 0 and 1 representing minimal and maximal diversity.

As previously reported [16], the clonality was  defined as 1—normalized Shannon entropy. The entropy eliminates the influence of sampling size, enabling comparison of clonotype frequency distributions across different samples. Clonality value ranges from 0 to 1. Values near 1 represent samples with one or a few predominant clones dominating the observed repertoire. Clonality values near 0 represent polyclonal samples.

Grouping of lymphocyte interactions by paratope hotspots version 2 (referred to as GLIPH2) was used to cluster TCRs into specificity groups predicted to share the same antigen specificity based on local motifs and/or global homology [17]. To identify T cell specificity groups, we used this algorithm to cluster TCRs through the web service: http:// 50. 255. 35. 37: 8080. The CD4/8 reference option was used, together with all other default options.

### RNA sequencing and differential expression analysis

RNA sequencing was performed at Shanghai Majorbio Bio-pharm Biotechnology Co., Ltd. (Shanghai, China). The raw paired end reads were trimmed and quality controlled by fastp [18] with default parameters. Then, the clean reads were separately aligned to reference genome with orientation mode using HISAT software [19]. Finally, the mapped reads of each sample were assembled by StringTie [20]. The RNA sequencing data were shown in Dataset S5.

Analysis of differential expression genes (DEGs) was performed using the DESeq2. The Kyoto Encyclopedia of Genes and Genomes (KEGG) and Gene Set Enrichment Analysis (GSEA) analysis were carried out by clusterProfiler and ggplot2 R package.

### Cell isolation and flow cytometry

Four tumor samples were prepared as previously described [21]. In brief, the freshly obtained surgical tumor samples were cut into approximately 1 mm^3^ pieces and incubated in DMEM + collagenase IV (1 mg/ml) + DNase (15 μg/ml) for 20 to 40 min at 37 °C. After tissue digestion, cell suspensions were subsequently passed through a 70-mm nylon cell strainer (Falcon). The remaining tissue pieces were mechanically dissociated using the back of a syringe over the cell strainer. All the cell samples were subsequently centrifuged for 10 min at 400 g. After centrifugation, cells were resuspended in 1 × phosphate-buffered saline (Invitrogen) with 1% fetal bovine serum. From single-cell suspensions, lymphocytes were isolated using Ficoll-Paque PLUS solution (Sigma-Aldrich).

Lymphocytes were stained with the following antibodies: APC/Cy7 anti-human CD45 (HI30), PE anti-human CD3 (OKT3), PE/Cy7 anti-human CD4 (OKT4), and PerCP/Cy5.5 anti-human CD8 (SK1) from Biolegend. CD4 + T cells and CD8 + T cells were sorted using the FACSAria III (BD).

### Immunohistochemistry and immunofluorescence

The formalin-fixed paraffin-embedded tissue specimens were sectioned continuously at four micrometers thick. For immunohistochemistry (IHC), slides were stained with anti-CD3 antibody (ab5690, Abcam), anti-CD4 antibody (ZM-0418, ZSJQ-BIO), anti-CD8 antibody (ZA-0508, ZSJQ-BIO), anti-programmed death ligand 1 (PD-L1) antibody (VENTANA-SP263, Roche), and anti-galectin 9 (GAL-9) antibody (ab227046, Abcam). The absolute number of positive lymphocytes for each antibody (CD3, CD4, and CD8) was analyzed in the 0.384 mm^2^ areas per tumor sample within the tumor center and counted using the Image J software. The IHC expression of PD-L1 was evaluated by percentage of membrane and/or cytoplasm stained and was considered positive with any staining in more than 5% of tumor cells [22]. The expression levels of GAL-9 were scored based on the staining intensity and percentage of positive cells [23]. The intensity of staining was scored as follows: absent, 0; weak, 1; moderate, 2; and strong, 3. The percentage of positive cells was scored as follows: 0%, 0; 1–10%, 1; 11–50%, 2; and 51–100%, 3 [24, 25]. Histological scores were calculated by multiplying both scores and high expression was defined as a score of 4–9. All stained slides were analyzed by two experienced pathologists blinded to other clinical information.

For immunofluorescence, slides were processed by hand using standard methods. Briefly, the primary antibodies were sequentially applied, followed by secondary antibody incubation and tyramide signal amplification. Between each round of staining, antigen retrieval was performed on the slides with heat-treatment. Finally, DAPI was applied to stain nuclei. The following antibodies were used to perform immunofluorescence on formalin-fixed paraffin-embedded samples: anti-CD8 antibody (ab178089, Abcam), anti-Ki-67 antibody (GB121141, Servicebio), anti-Granzyme B (GZMB) antibody (ab208586, Abcam), anti-programmed cell death protein 1 (PD-1) antibody (ab52587, Abcam), anti-LAG-3 antibody (ab209236, Abcam), and anti-Tim3 antibody (ab241332, Abcam). The slides were scanned with the PhenoImager Fusion system (Akoya) and viewed with the Phenochart software 1.2.0.

### Statistical analysis

Graphpad Prism 8.0 (GraphPad Software, San Diego, CA) was used for statistical and graphical analyses. Comparisons between any two groups were analyzed using the Wilcoxon matched pairs test, Student *t* test or unpaired two-sided Student *t* test with Welch’s correction, and *P* < 0.05 was considered statistically significant. Correlations between variables were analyzed using Spearman’s rank test.

## Results

### Comparable *TRBV* and *TRBJ* gene segments usage patterns and CDR3 length distribution between tumor and adjacent normal tissues

**Fig. 1:**
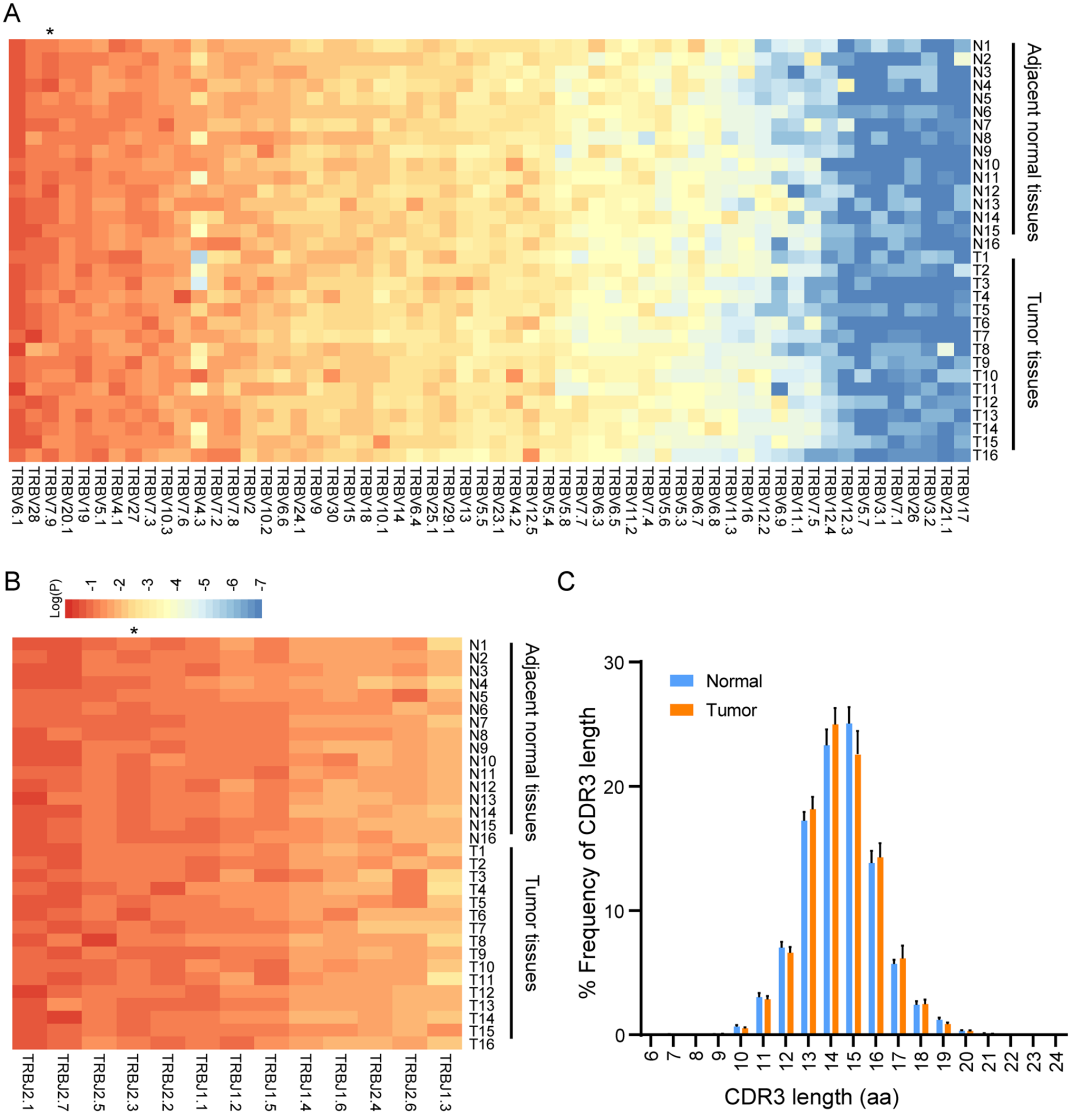
Comparing the usage of *TRBV* and *TRBJ* genes and length distribution of CDR3 sequences between tumor tissues and paired adjacent normal tissues. Heat map of the usage frequency of **A** *TRBV* gene and **B** *TRBJ* gene between tumor tissues and normal tissues. Statistics are based on the paired Student *t* test. *P < 0.05. *TRBV* gene or *TRBJ* gene frequencies were significantly lower (black star) in tumor tissues than in paired adjacent normal tissues. **C** Comparison of CDR3 aa sequence length distribution between tumor and normal tissues. Data represent the mean ± SEM of frequencies. T, tumor tissue; N, normal tissue

TCR sequencing was conducted on 22 tumor tissues and 16 paired adjacent normal tissues from 22 patients with PSCC, whose TCRβ chain sequence profiles are shown in  Dataset S3. We obtained 2,287,097–1,0261,153 productive reads in tumor tissues and 499,799–7,111,217 productive reads in paired adjacent normal tissues. By comparing T cell receptor beta-variable (*TRBV*) and joining (*TRBJ*) genes in TCR repertoires, we found that *TRBV*/*TRBJ* gene usage was similar between tumor and paired adjacent normal tissues (Fig. 1A, B). In both tissues, the most frequent *TRBV* gene segments were *TRBV6.1*, *TRBV28*, *TRBV7.9*, *TRBV20.1*, and *TRBV19*. In addition, the most frequent *TRBJ* gene segments were *TRBJ2.1*, *TRBJ2.7*, *TRBJ2.3*, *TRBJ2.5* and *TRBJ2.2*. *TRBV7.9* (*p* = 0.0101*)* and *TRBJ2.3* (*p* = 0.0309) gene segments usage was lower in tumor tissues, as compared with normal tissues. Since distribution of CDR3 sequence length provides a general view of the TCR repertoire composition [26, 27], we also compared the length distribution of CDR3 amino acid (aa) sequences between tumor and normal tissues. Similar CDR3 aa length distributions were observed in tumor and normal tissues, with CDR3 lengths ranging from 10 to 20 aa and a peak frequency of 15 aa (Fig. 1C). Overall, the usage pattern of *TRBV* or *TRBJ* genes and CDR3 aa length distribution were comparable between tumor and matched adjacent normal tissues.

### Limited overlap of TCR repertoire between tumor and adjacent normal tissues

**Fig. 2:**
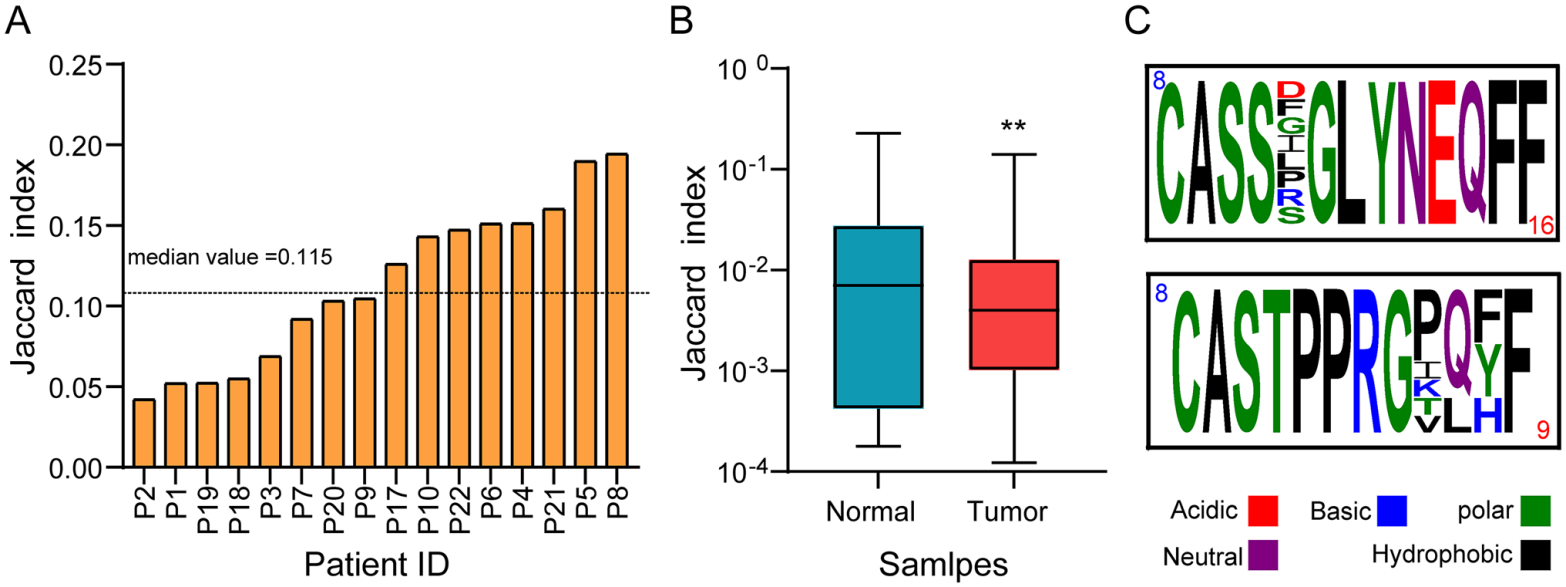
Inter-tissue and intra-tissue overlap of TCR repertoires. **A** TCR repertoire overlap between tumor and paired adjacent normal tissue for each patient with PSCC was calculated using the Jaccard index. **B** TCR repertoire overlap between tissues of different individuals was calculated by the Jaccard index. **C** Representative motifs found in the 18,800 clonally expanded specificity groups. Graphics were generated with Weblogo. The red number on top left of the box reveals the total number of patients who have convergent TCRs. Blue number on bottom right of box shows the total number of different TCRs that contain that motif. Statistics were based on the Wilcoxon matched pairs test. ** *P* < 0.001

To assess the overlap of the TCR repertoire between tumor and adjacent normal tissues, we calculated the Jaccard index, whose value ranges from 0 (no overlap) to 1 (identical). We found a limited overlap of TCR repertoire between tumor and paired adjacent normal samples for 16 patients with PSCC. The median Jaccard index was 0.115, ranging from 0.042 to 0.194 (Fig. 2A). In addition, the overlap of TCR repertoire among 16 adjacent normal tissues or among 16 tumor tissues was limited, thus evidenced by extremely low Jaccard index for adjacent normal tissues (median: 0.007, range: 0.228 to 1.79 × 10^–4^) and tumor tissues (median: 3.09 × 10^–3^, range: 0.141 to 1.23 × 10^–4^) (Fig. 2B). However, significantly (*p* = 0.0049) higher overlap of TCR repertoire was observed in normal tissues than that in tumor tissues (Fig. 2B), indicating the intratumor heterogeneity of PSCC. Taken together, our results suggest that TCR repertoires were different between tumor and adjacent normal tissues and highly varied among individuals.

By using the grouping of lymphocyte interactions with paratope hotspots 2 to cluster the TCRβ CDR3 amino acid sequences (top 3000 TCRs in each patient) based on local motifs and/or global homology [17], we further analyzed the antigen expression patterns of these PSCC tumors and identified 18,800 shared specificity clusters. Every cluster consisted of a single or maximum eight unique sequences that were detected in more than one patient (representative motifs are shown in Fig. 2C and detailed motifs are shown in Dataset S4a), suggesting that there were shared antigen expression patterns between PSCC tumors. Furthermore, we analyzed the TCR-antigen specificities of these intratumoral expanded clonotypes by using VDJdb, a curated database of known TCR-antigen specificities [28]. Among the 27,604 unique clonotypes, only 742/27,604 (2.69%) of them matched previously reported sequences to recognize known epitopes, such as common cytomegalovirus and influenza A (Dataset S4b), indicating that most of the highly expanded TCRs might be tumor-specific.

### Higher TCR clonality and lower TCR diversity in tumor tissues compared with normal penile tissues

**Fig. 3:**
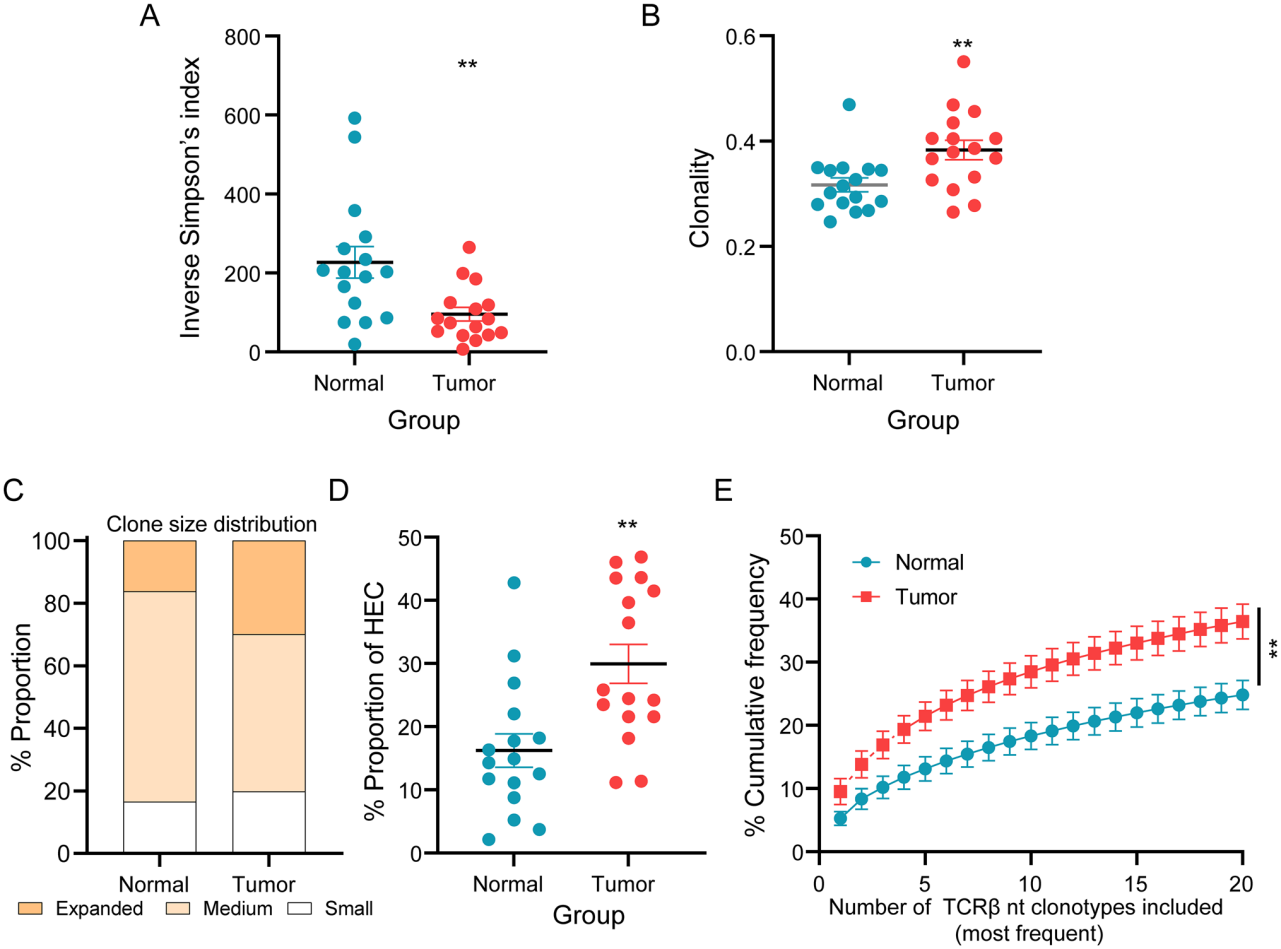
Clonal expansion and diversity of T cell repertoire in tumor and normal tissues. **A**, **B** Comparison of the **A** inverse Simpson’s diversity index and **B** clonality index between tumor tissues and paired adjacent normal tissues. **C** Distribution of TCR clonotypes of different sizes. Small, 0 < *x* ≤ 0.1%, medium 0.1% < *x* ≤ 1%, and expanded, *x* > 1%. TCR clonal size was normalized by the total number of TCR nt sequences. **D** Comparison of the percentage of highly expanded clonotypes between tumor and normal tissues. **E** Cumulative frequencies occupied by the 20 most frequent clonotypes for each tissue type. Data were calculated for sequences from 16 samples per tissue type. Data in the bar graphs represent means ± SEM. Statistics were based on the Wilcoxon matched pairs test. ** P < 0.001; HEC, highly expanded clonotypes; nt, nucleotide

TCR diversity and clonality have been reported to be important in predicting prognosis and response to immunotherapy [11, 13, 29, 30]. Therefore, we compared TCR diversity using inverse Simpson’s diversity index, and TCR clonality using the normalized Shannon entropy index between tumor tissues and adjacent normal tissues. We found that tumor tissues had significantly lower diversity (*p* = 0.0027, Fig. 3A) and higher clonality (*p* = 0.0067, Fig. 3B), as compared with adjacent normal tissues. Furthermore, we assessed the frequency distribution of TCR clonotypes in the whole repertoire. We categorized TCR clonotypes into three groups based on the following frequencies: > 1% = expanded, 0.01% to 1% = medium, and ≤ 0.1% = small (Fig. 3C). TCR repertoire of tumor samples was highly skewed with a significantly (*p* = 0.0063) higher fraction of TCRs occupied by highly expanded clonotypes, compared with normal tissues (Fig. 3D). In addition, we assessed intratumoral TCR repertoires as evidence of clonal expansion and used the cumulative frequency curves to measure the 20 most abundant clonotypes (top20 clonotypes) (Fig. 3E). The cumulative frequency of the top20 clonotypes in tumor tissues accounted for approximately 36% of the whole repertoire and was significantly (*p* = 0.0063) higher than that of adjacent normal tissues (Fig. 3E). These results demonstrate that diversity and clonality of the T cell repertoire in tumor samples from patients with PSCC are clearly different from that of normal tissues.

### Correlation between intratumoral TCR repertoire and clinical characteristics

**Fig. 4:**
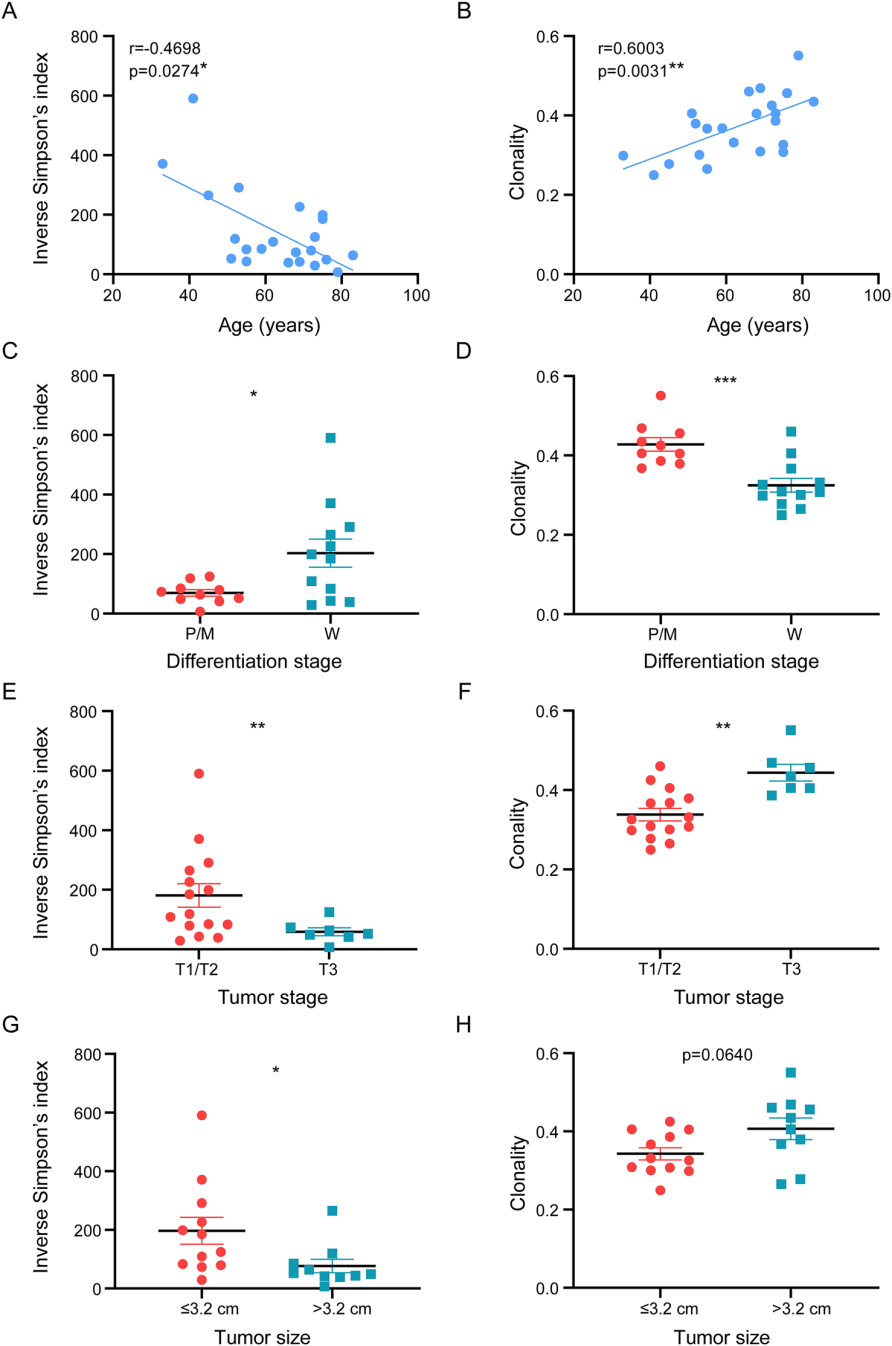
Relationship between TCR repertoire and individual characteristics. **A**, **B** Correlation between age and **A** TCR diversity or **B** clonality. **C**, **D** Comparison of **C** TCR diversity or **D** clonality between different pathological stages of PSCC. **E**, **F** Comparison of **E** TCR diversity or **F** clonality index based on tumor stages of patients with PSCC. **G**, **H**. Comparison of **G** TCR diversity or **H** clonality between patients with a primary tumor diameter greater than 3.2 cm and patients with a primary tumor diameter less than 3.2 cm (median tumor size). Data in the bar graphs represent means ± SEM. The Spearman’s rank test and unpaired two-sided Student *t* test with Welch’s correction were used for analysis. *P < 0.05, ** P < 0.001, *** P < 0.0001. P, poorly; M, moderately; W, well

We also investigated the association of TCR features with clinical data, such as age, pathological type, and tumor stage and size. We observed that TCR diversity and clonality were affected by age (Fig. 4A, B), as previously reported [31, 32]. TCR diversity in tumor samples negatively correlated with age (*r* = − 0.4698, *p* = 0.0274), whereas TCR clonality positively correlated with age (*r* = 0.6003, *p* =0.0031 ). However, we did not observe a correlation between TCR repertoire and HPV status (diversity, *p* = 0.9312; clonality, *p* = 0.5150) or histological subtypes (diversity, *p* = 0.7568; clonality, *p* = 0.5472; Fig. S1).

In addition, we compared the TCR repertoire among patients with different pathological grades (poorly, moderately, and well-differentiated), and different tumor stages (T1/2 and T3) sizes. Lower diversity and higher clonality were found in PSCC patients with poorly and moderately differentiated tumors (diversity, *p* = 0.0179, Fig. 4C; clonality, *p* = 0.0004, Fig. 4D) and in PSCC patients with high T stages (diversity,* p* = 0.0093, Fig. 4E; clonality, *p* = 0.0015, Fig. 4F). When the tumor diameter was larger than 3.2 cm, diversity was significantly (*p* = 0.0323) lower (Fig. 4G), and clonality was slightly higher (*p* = 0.0640; Fig. 4H). Taken together, these data demonstrate that patients with advanced PSCC (poor differentiation, high stages, and larger sizes) have lower TCR diversity and higher TCR clonality.

### Differentially expressed genes between low-clonality and high-clonality groups

**Fig. 5:**
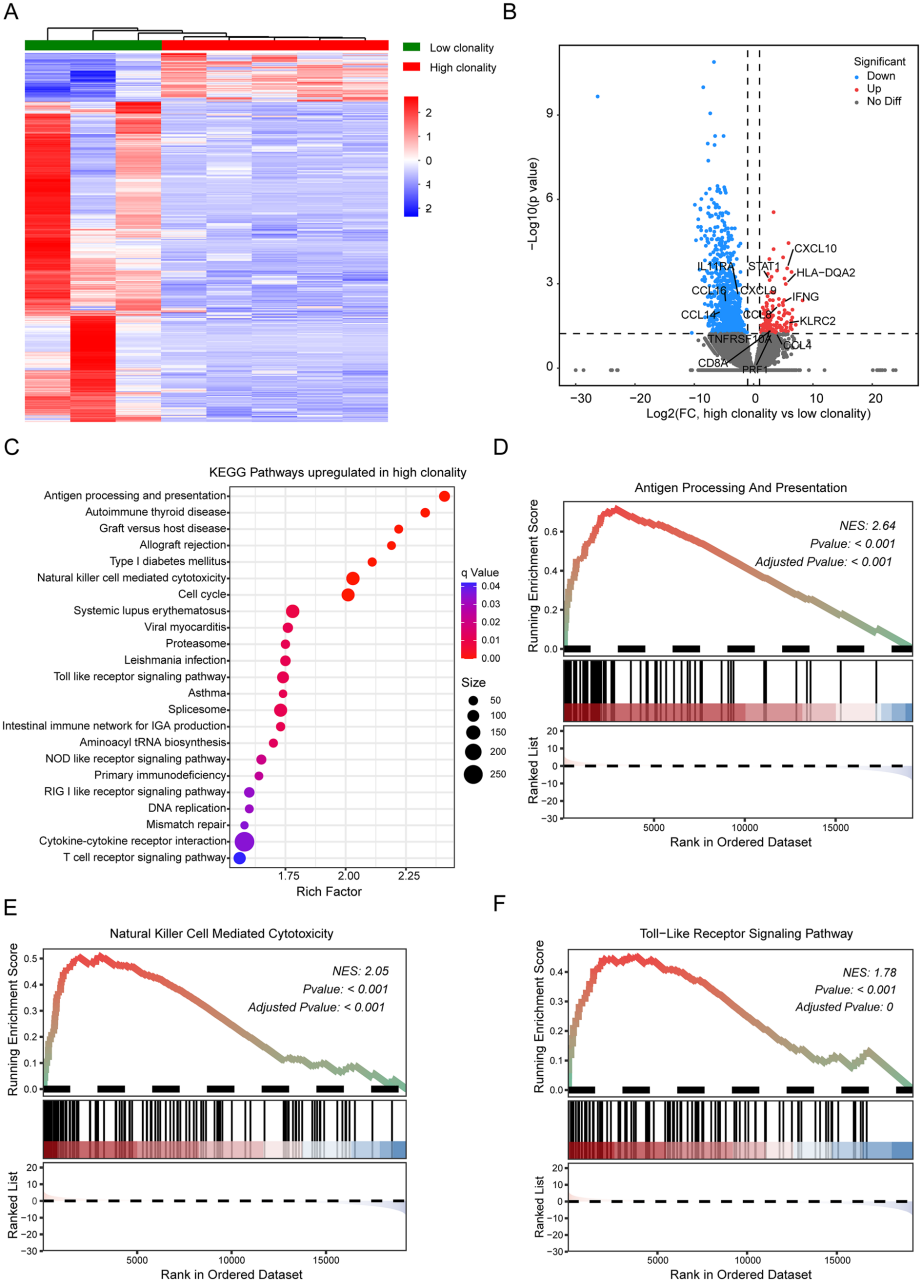
Transcriptomic analyses revealed immune activation signatures in patients with high clonality. **A** Comparing hierarchical clustering of DEGs between patients with “high-clonality” (red) and “low-clonality” (blue). **B** Volcano plot of significant DEGs between high- and low-clonality groups. **C** KEGG pathway analysis of upregulated genes in the high-clonality group. The vertical axis represents the pathway category, and the horizontal axis represents the enrichment score [− log(*q*-value)] of the pathway. Significantly enriched KEGG pathways (*q* value < 0.05) are presented. **D**–**F** Gene set enrichment analyses revealed that **D** antigen processing and presentation, **E** natural killer cell-mediated cytotoxicity, and **F** toll-like receptor signaling pathway were significantly enriched in the high-clonality group

Since different TCR clonalities correlated with the malignant behavior of penile cancer, we determined the molecular characteristics underlying different TCR clonalities. According to the cutoff value of 0.373, we divided our cohort into “high-clonality” and “low-clonality” groups. We identified 1151 differentially expressed genes (DEGs) in tumor tissues between these two groups, using the whole transcriptome profile (Fig. 5A and S2). The major histocompatibility complex antigen-presenting genes, such as HLA-DQA2 and HLA-C, cytotoxic effector genes, such as KLRC2, PRF1, and IFNG, were significantly higher in the high-clonality group (Fig. 5B), compared with the low-clonality group. Furthermore, KEGG pathway enrichment analysis confirmed that upregulated DEGs in the high-clonality group were significantly enriched in immune-related processes, such as antigen processing and presentation, killer cell-mediated cytotoxicity, and toll-like receptor signaling pathway (Fig. 5C), which were further supported by GSEA results (Fig. 5D–F). We also observed higher expression levels of CXCL9, CXCL10, CXCL11, CCL4, and CCL8 and lower expression level of the chemokines CCL14 and CCL16 in the high-clonality group (Fig. 5B). CXCL9, 10, and 11/CXCR3 axis have been reported to regulate migration, differentiation, and activation of immune cells, including CD8 + T cells [33]. Additionally, the expression levels of CD8-alpha and STAT1 genes were significantly upregulated in the high-clonality group (Fig. 5B), suggesting an association between high clonality and gene expression patterns in CD8 + T cells. Collectively, our data indicate that the high clonality of TCR in PSCC might be attributed to CD8 + T cells.

### Correlation between TCR clonality and CD8 + T cells

**Fig. 6:**
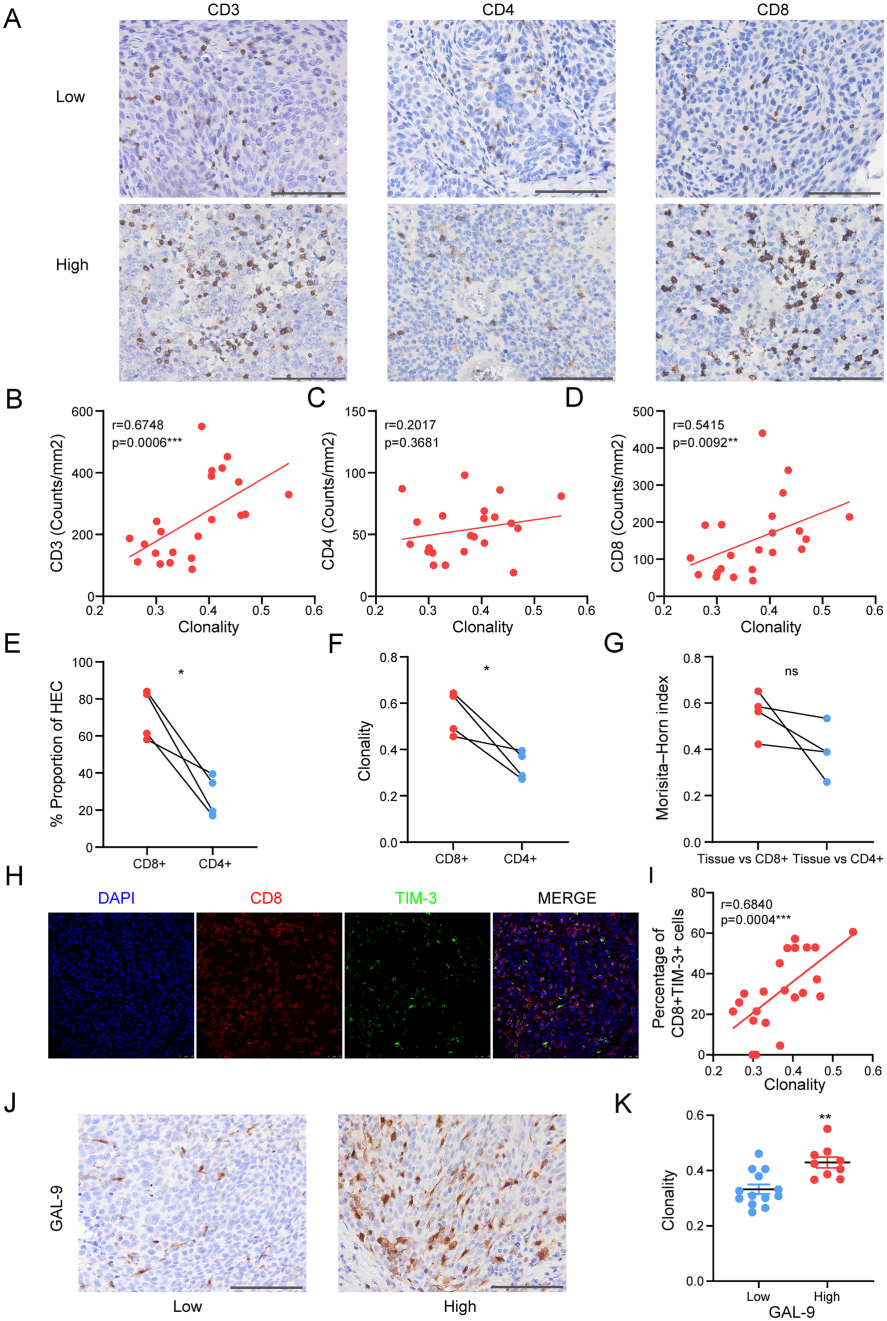
TCR clonality was associated with exhausted CD8 + T cells. **A** Representative IHC images of different subsets of T lymphocytes from penile tumor tissues (from left to right): CD3 + T cells, CD4 + T cells, and CD8 + T cells between the low-clonality (up) and high-clonality (bottom) groups. Scale bar = 200 μm. Magnification, 400X. **B**–**D** Correlation between TCR clonality and **B** CD3 density (*n* = 22), **C** CD4 density (*n* = 22), and **D** CD8 density (*n* = 22) measured by IHC. **E** Percentage of high-frequency clonotypes within the sorted CD4 + T cells and CD8 + T cells. **F** Clonality index of sorted CD4 + T cells and CD8 + T cells. **G** Similarities of TCR repertoire for each patient analyzed by the Morisita–Horn index between repertoire of the whole tissue and the CD4 + or CD8 + population. **H** Immunofluorescence staining of CD8 and TIM-3 in one patient. Scale bar = 25 μm. **I** Correlation between TCR clonality and the percentage of TIM-3 + CD8 + cells among total CD8 + cells. **J** Representative cases were showing GAL-9 expression. Left, low GAL-9 expression; right, high GAL-9 expression. Scale bar = 200 μm. Magnification, 400X. (K) TCR clonality in GAL-9^hi^ tumors and GAL-9^lo^ tumors. Data represent means ± SEM. The Spearman’s rank test, paired Student *t* test, and unpaired two-sided Student *t* test with Welch’s correction were used for analysis. ns, not significant; * *P* < 0.05, ** P < 0.001, *** P < 0.0001

To further substantiate the roles of CD8 + T cells in TCR clonality, we analyzed the relationship between T cell infiltration and TCR clonality using IHC staining for CD3 (T cells), CD4 (helper T cells), and CD8 (cytotoxic T cells) in PSCC tumor samples (Fig. 6A). Consistent with previous observations [34], we found that CD8 + T cells were the predominant subtype of T cells in penile tumor samples (*p* = 0.0001, Fig. S3A), with an average CD8:CD4 ratio of 3.11 (ranging from 0.43 to 9.20) (Fig. S3B). We also evaluated the relationship between cell number in different subtypes of T cells and tumor TCR repertoire, and found that TCR clonality correlated only with the cell numbers of CD3 + (*r* = 0.6748; *p* = 0.0006; Fig. 6B) and CD8 + cells (*r* = 0.5414; *p* = 0.0092; Fig. 6D), but not with CD4 + cells (*r* = 0.2017; *p* = 0.3681; Fig. 6C), suggesting that T cell clonality in PSCC may be mainly driven by the clonal expansion of CD8 + T cells.

Next, we sorted T cells from tumor samples of four patients with advanced PSCC using flow cytometry (Fig. S3C) and investigated the repertoires of sorted CD4 + T cells and CD8 + T cells for each patient (Fig. S3D). In all patients, we observed that sorted CD8 + T cells contained a higher proportion of highly expanded clonotypes, compared with sorted CD4 + T cells (*p* = 0.0181, Fig. 6E). Consistently, the clonality of CD8 + T cell repertoire was significantly higher than that of paired CD4 + T cells (*p* = 0.0324, Fig. 6F). Furthermore, there was a clear trend that repertoires from whole tissues were more similar to those from sorted CD8 + T cells rather than those from CD4 + T cells although the differences were not statistically significant (*p* = 0.1436, Fig. 6G). These results indicate that most clonal expansions associated with malignant penile tumors were within the CD8 + T cell lineage.

To investigate the phenotypic characteristics of CD8 + TILs, which contribute to TCR clonality, we performed immunofluorescence to analyze the phenotypes of CD8 + TILs. We found that CD8 + TILs in PSCC tumors possessed low proliferation potential and cytotoxicity, evidenced by few (median: 4.9%) Ki-67 + CD8 + cells and few (median: 2.3%) GZMB + CD8 + cells, respectively (Fig. S4A, B and Dataset S6). We observed that the majority of CD8 + TILs in PSCC tumors were exhausted, as shown by high percentages of TIM-3 + CD8 + (median: 30.3%) and LAG-3 + CD8 + (median: 30.4%) cells among CD8 + TILs (Figs. 6H and S4D and Dataset S6). Of the tumors examined, 9 (40.9%) had high Gal-9 expression, and 15 (68.2%) were positive for PD-L1 expression (Figs. 6J and S4F and Dataset S6). Noteworthy, PD-1 + CD8 + cells were less frequently observed in our PSCC cohort (Fig. S4C and Dataset S6), which needs to be investigated. More importantly, we found that TCR clonality positively correlated with exhausted CD8 + cells, including TIM-3 + CD8 + cells (*r* = 0.6840; *p* = 0.0004; Fig. 6I) and LAG-3 + CD8 + cells (*r* = 0.5878; *p* = 0.0040; Fig. S4E). In addition, PSCC tumor cells with high expression of T cell inhibitory ligands were associated with high TCR clonality (Figs. 6K and S4G). Taken together, these results suggest that exhausted CD8 + TILs with high expression levels of TIM-3 and LAG-3 might contribute to TCR clonality in PSCC tumors.

## Discussion

The adaptive immune system is essential in fighting diseases, especially cancer. It is widely accepted that the TCR repertoire is shaped by antigen engagement and altered in the context of the disease [35]. Although important roles of TCR repertoire have been revealed in different types of tumors [32, 36–38], our study is the first to describe the characteristics of TCR repertoire in patients with PSCC. Using high-throughput TCR sequencing, we showed the following aspects: ① the overlap of TCR repertoire between tumor and normal tissues was very limited, ② lower TCR repertoire diversity and higher clonality were observed in PSCC tissues, as compared with normal tissues, ③ TCR repertoire in PSCC correlated with clinicopathologic features; specifically, increased clonality and reduced diversity were observed in advanced tumors (tumors with poor differentiation, high stages, and larger sizes), ④ PSCC tumors with high TCR clonality showed hyperactivation of immune-related genes, and highly expanded clonotypes in tumor samples mainly originated from CD8 + T cells, ⑤ exhausted CD8 + TILs with high expression levels of TIM-3 and LAG-3 might contribute to the TCR clonality in PSCC tumors.

TCR repertoire has been linked to tumor malignant behavior and prognosis [12]. Yang et al*.* showed that patients with advanced lung cancer have higher TCR clonality, which was associated with tumor stage [39]. Similarly, Sanz-Pamplona et al*.* reported that patients with higher TCR clonality have poorer overall prognosis in stage II microsatellite stable colorectal cancer [40]. In contrast, Lecuelle et al*.* found that high clonality is significantly associated with better survival in high-grade serous ovarian carcinoma [41]. Conflicting findings related to the role of TCR clonality in different tumors might be due to immune microenvironment variations, which require further studies on specific tumor types. We found that TCR clonality was higher in PSCC tumor tissues compared with adjacent normal tissues. Moreover, higher clonality of TCR repertoire correlated with advanced penile cancer (poor differentiation, high stages, and larger sizes).

Previous studies have shown the key role of TCR clonality in predicting the therapeutic response to immunotherapy. Studies on melanoma found that patients with high clonal expansion (clonality) of T cells have better clinical responses to ICB [42, 43]. Patients with non-small lung cancer and increased PD-1 + CD8 + TCR clonality after ICB had longer progression-free survival than those with decreased PD-1 + CD8 + TCR clonality [11]. However, we could not investigate the relationship between TCR clonality and the therapeutic response of patients with PSCC due to the following issues: ① most patients did not receive drugs; ② the small size of the sample and ③ the short duration of follow-up period. In our study, we demonstrated the association between TCR clonality and the number of CD8 + T cells infiltrating into PSCC tissue. Since higher CD8 + T cell infiltration is associated with better clinical prognosis in patients with PSCC [6, 44], TCR clonality may be an important biomarker for predicting the therapeutic response to immunotherapy in patients with PSCC, which needs further investigation.

TCR clonality reflects the presence of the highly cloned T cells in the tumor microenvironment, where CD4 + T cells and CD8 + T cells play important roles in the anti-tumor immune responses [45]. Zhang et al*.* found that tumor-infiltrating CD4 + T cells exhibited specific clonal expansion driven by tumor-associated antigens in clear cell renal cell carcinomas [46]. T cell clonality was associated with CD8 + T cells in non-small cell lung cancer and T cell clonality positively correlated with GZMB and IFN-γ expression [14]. Our findings indicated that T cell clonality of PSCC might be attributed to CD8 + T cells. We demonstrated that ① Compared with the low-clonality subgroup, higher expression levels of CD8-alpha, STAT1 (related to expansion and memory formation of CD8 + T cells), CXCL9, CXCL10, and CXCL11 (chemokines important for CD8 + T cell migration and differentiation) were found in the high-clonality subgroup and ② TCR sequencing of sorted T cells showed that most expanded clonotypes in patients with PSCC were of the CD8 T cell lineage.

Several clinical trials have been recently conducted to assess the role of ICB in penile cancer [5]. In our study, we found high infiltration of exhausted CD8 + cells in PSCC tumors. We then hypothesized that PSCC tumors might respond to ICB therapy. However, we detected a low PD-1 expression on CD8 + T cells in our cohort, despite most tumors were PD-L1 positive. Therefore, the effects of targeting PD-1/PD-L1 on PSCC require further investigation. We also found a high percentage of TIM-3 + cells among CD8 + TILs and high GAL-9 expression in almost half of PSCC tumors, which correlated with high TCR clonality. Previous studies have shown that TIM-3-GAL-9 pathway plays an important role in suppressing anti-cancer immune surveillance, whose blockade stimulates anti-tumor immune responses [47–49]. Therefore, our results suggested that PSCC patients with high TCR clonality were more likely to benefit from ICB therapies targeting TIM-3/GAL-9, which needs to be further investigated.

Our study has the following limitations: ① PSCC is a very rare disease and we evaluated a small cohort of patients, ② multi-region sampling would be needed to fully capture the intratumoral heterogeneity of geographically distinct tumor regions, and ③ additional experimental and clinical efforts are needed to establish the correlation between the TCR repertoire and prognosis of patients with PSCC.

## Conclusions

In conclusion, we comprehensively assessed the TCR repertoire in tumor and adjacent normal tissues from patients with PSCC. Our findings suggest that TCR repertoire of tumor tissues in patients with PSCC was significantly different from that of normal tissues. Moreover, high TCR repertoire clonality (low diversity) was associated with tumor malignant behavior in patients with PSCC. Tumor tissues with high TCR clonality showed hyperactivation of immune-related genes, and those highly expanded clonotypes in tumor samples mainly originated from CD8 + T cells. More importantly, exhausted CD8 + TILs with high expression levels of TIM-3 and LAG-3 might contribute to TCR clonality in PSCC tumors. Nevertheless, further studies with larger sample sizes and more detailed clinical parameters are needed to determine TCR clonality as a potential biomarker for prognosis and therapeutic response to ICB, targeting TIM-3 or LAG-3 in patients with PSCC.

### Supplementary Information

Below is the link to the electronic supplementary material.

Supplementary file1 (TIF 2983 KB)

Supplementary file2 (TIF 1890 KB)

Supplementary file3 (TIF 5286 KB)

Supplementary file4 (TIFF 25390 KB)

Supplementary file5 (XLSX 11 KB)

Supplementary file6 (XLSX 13 KB)

Supplementary file7 (XLSX 14 KB)

Supplementary file8 (XLSX 14305 KB)

Supplementary file9 (XLSX 12 KB)

Supplementary file10 (XLSX 12 KB)

Supplementary file11 (DOCX 1621 KB)

## Data Availability

The raw data in this study were freely available in Genome Sequence Archive (GSA) database with accession numbers HRA006203 and HRA006205.

## Abbreviations

aa: Amino acid
CDR3: Complementarity determining region 3
DEGs: Differentially expressed genes
GAL-9: Galectin-9
GZMB: Granzyme B
HPV: Human papillomavirus
ICB: Immune checkpoint blockade
LAG-3: Lymphocyte activation gene 3
PD-1: Programmed cell death protein 1
PD-L1: Programmed death ligand 1
PSCC: Penile squamous cell carcinoma
TCR: T cell receptor
TRBJ: T cell receptor beta-joining
TRBV: T cell receptor beta-variable
TIM-3: T cell immunoglobulin mucin receptor 3
TIL: Tumor-infiltrating lymphocyte
