# Supplementary figures and images for: Characterization of T cell receptor repertoire in penile cancer
Source: Cancer Immunol Immunother. 2024 Jan 27;73(2):24. doi: 10.1007/s00262-023-03615-z (PMC10822009; doi:10.1007/s00262-023-03615-z)

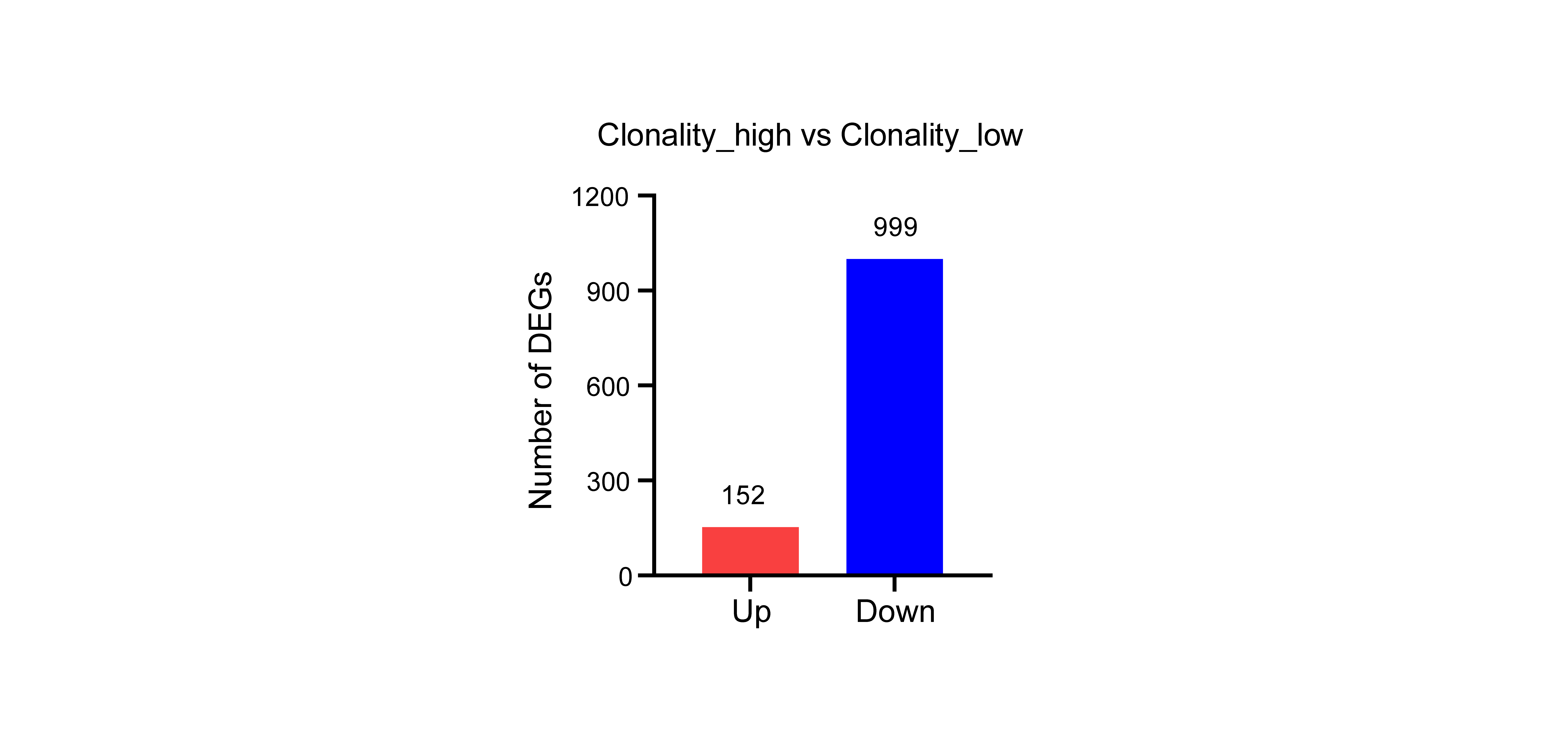

Supplement: Supplementary file 2 — Supplementary file2 (TIF 1890 KB) [file 262_2023_3615_MOESM2_ESM.tif]

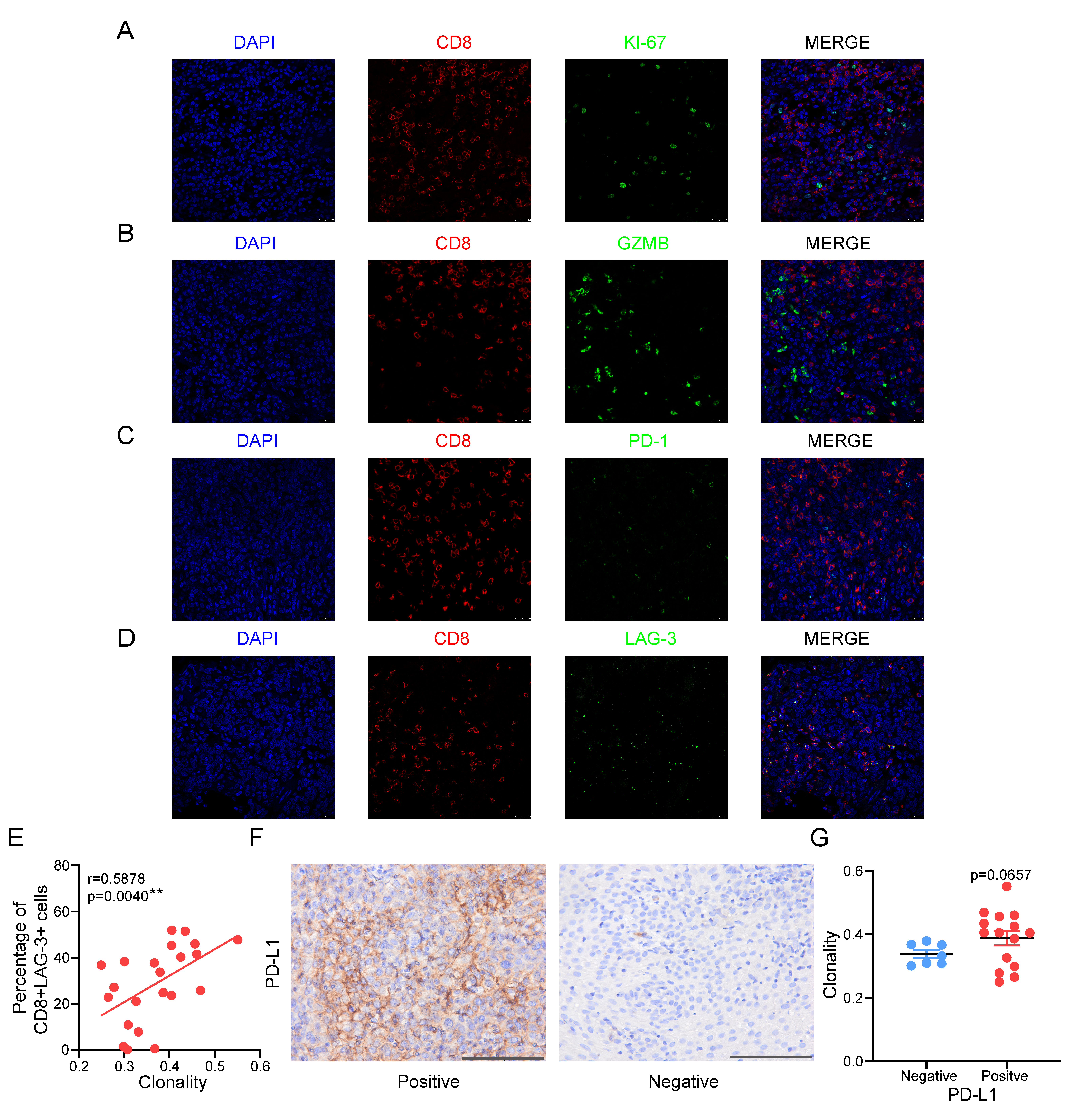

Supplement: Supplementary file 4 — Supplementary file4 (TIFF 25390 KB) [file 262_2023_3615_MOESM4_ESM.tif]
