# Supplementary material for: Characterization of T cell receptor repertoire in penile cancer
Source: Cancer Immunol Immunother. 2024 Jan 27;73(2):24. doi: 10.1007/s00262-023-03615-z (PMC10822009; doi:10.1007/s00262-023-03615-z)

**Figure S1.** **Correlation between TCR repertoire and HPV status and histologic subtype.**

(A, B) Comparison of (A) diversity and (B) clonality between HPV− and HPV+ patients. (C, D) Comparison of (C) diversity and (D) clonality between usual and other histological subtypes. Statistical analyses were based on the unpaired two-sided Student *t* test with Welch’s correction. ns, not significant.


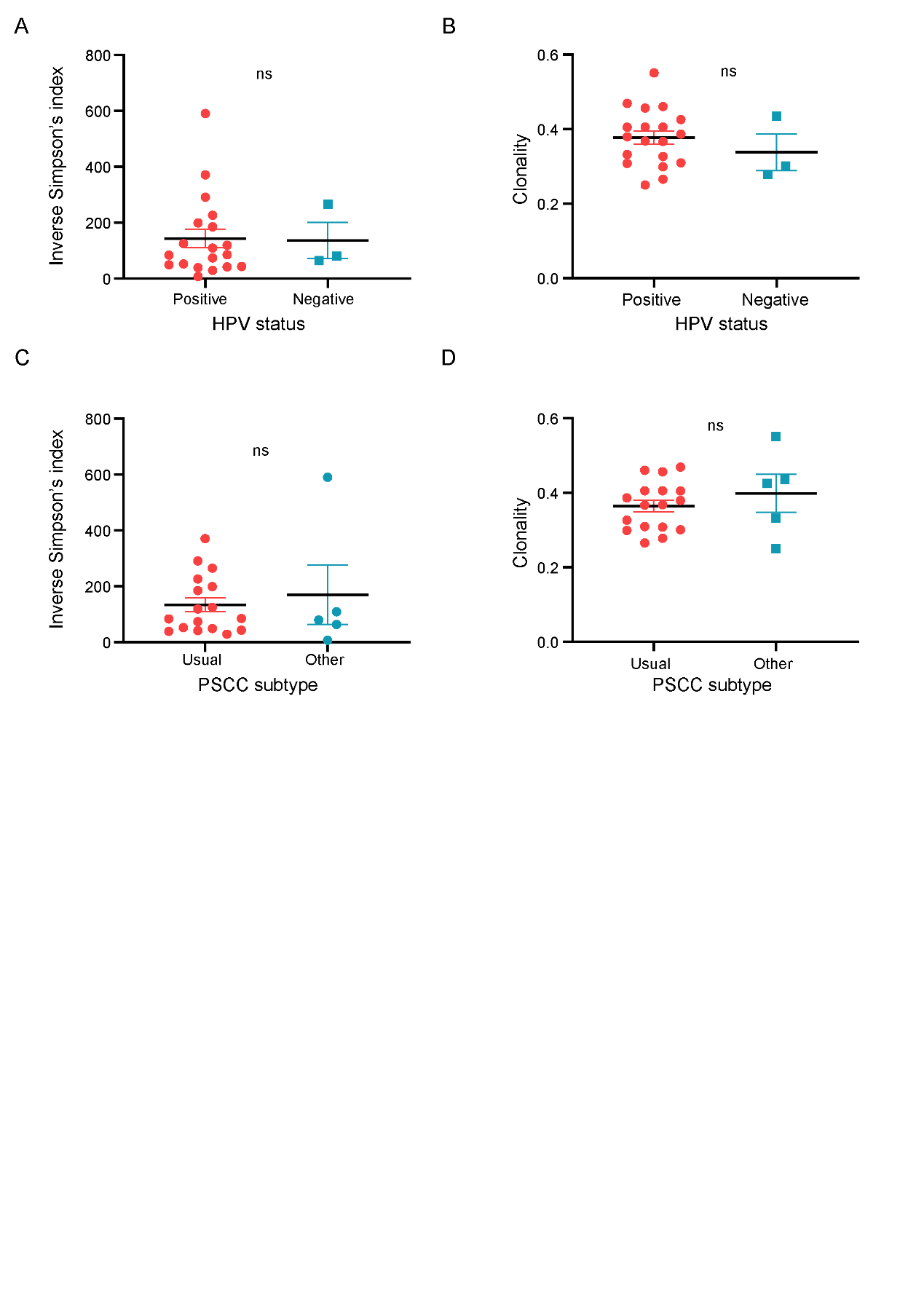


**Figure S2. Comparison of DEGs between the low- and high- clonality groups.**


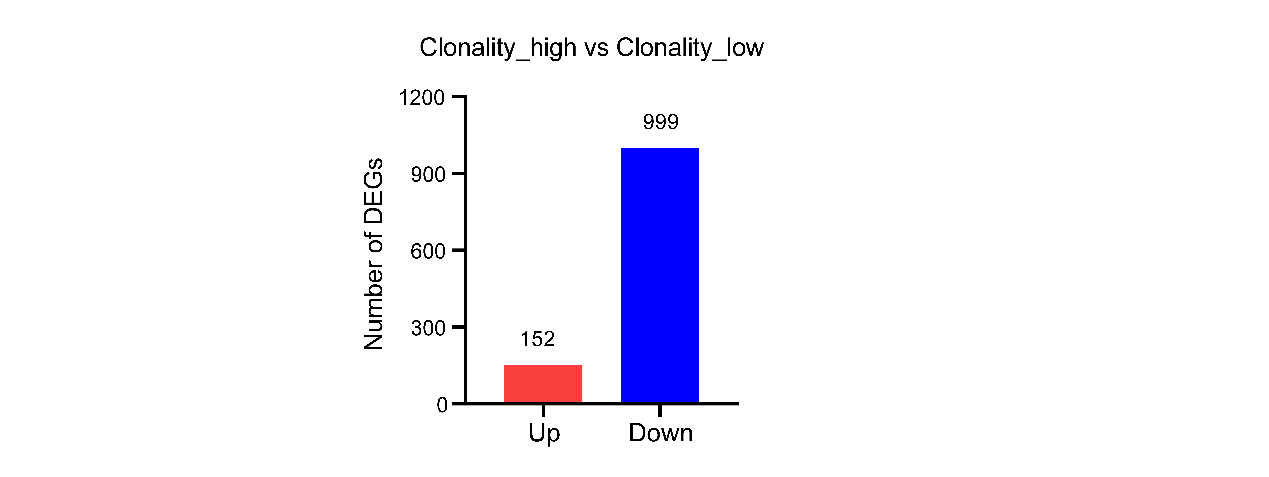
Number of DEGs between patients with “high-clonality” and “low-clonality”.

**Figure S3. CD8+ T cells are major components of the tumor microenvironment in PSCC.**

(A) Number of CD4+ T cells and CD8+ T cells per mm^2^ in each tumor sample. (B) CD8:CD4 ratio in each tumor sample. (C) Representative flow cytometric analysis of T cell subsets from tumor samples. (D) Distribution of TCR clonotypes of varied sizes. Statistics were based on the paired Student *t* test. ns, not significant; *** *P* < 0.0001.


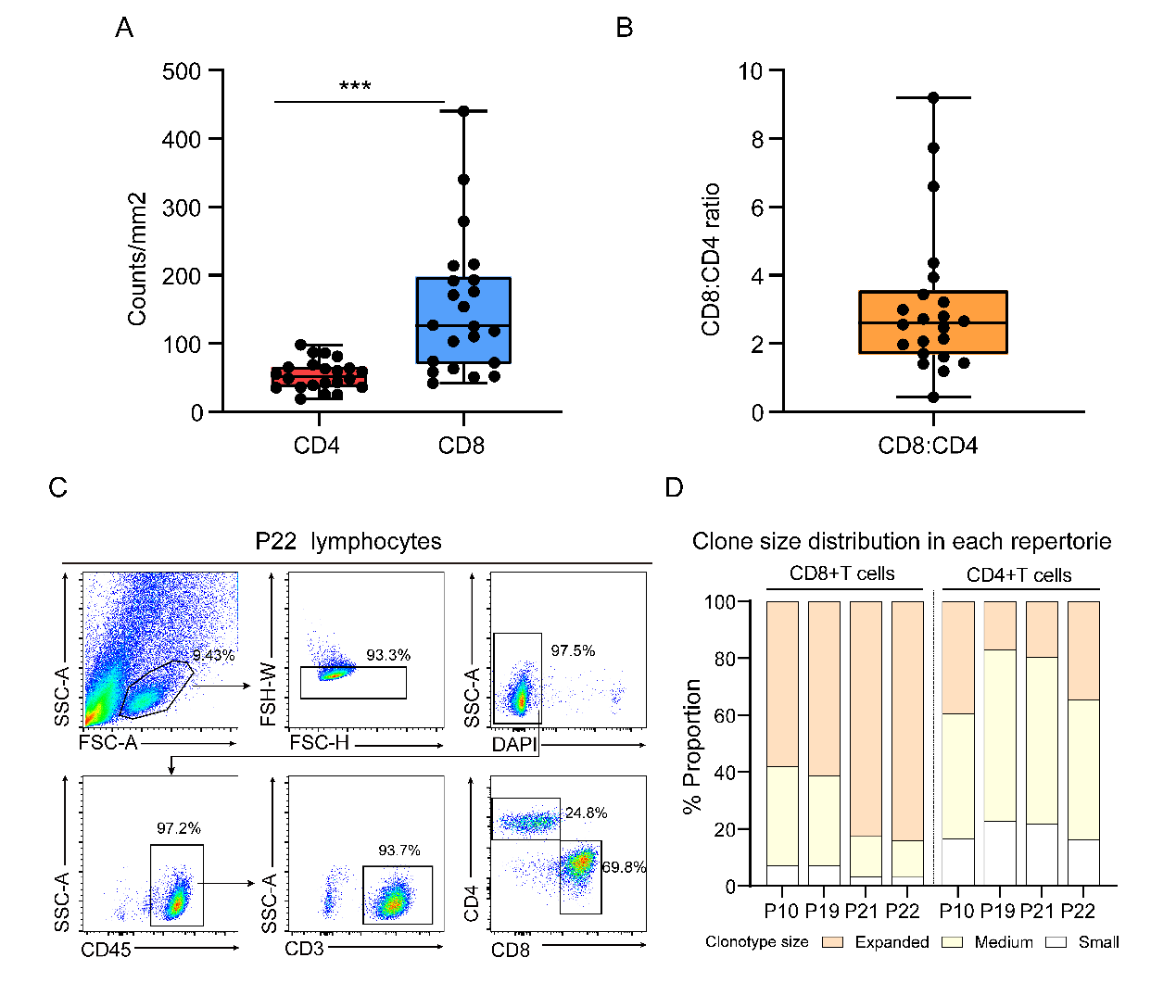


**Figure S4. Phenotypic characteristics of CD8+ TILs and tumor cells in PSCC tumors.**

(A, D) Immunofluorescence staining of (A) CD8 and Ki-67, (B) CD8 and GZMB, (C) CD8 and PD-1 and (D) CD8 and LAG-3. Scale bar= 25 μm. (E) Correlation between TCR clonality and the percentage of LAG-3+ CD8+ cells among total CD8+ cells. (F) Representative cases were showing PD-L1 expression. Left, low PD-L1 expression; right, high PD-L1 expression. Scale bar = 200 μm. Magnification, 400X. (G) TCR clonality in PD-L1^pos^ tumors and PD-L1^neg^ tumors. Data represent mean ± SEM. The Spearman’s rank test and unpaired two-sided Student *t* test with Welch’s correction were used for analysis. * *P* < 0.05, ** *P* < 0.001.


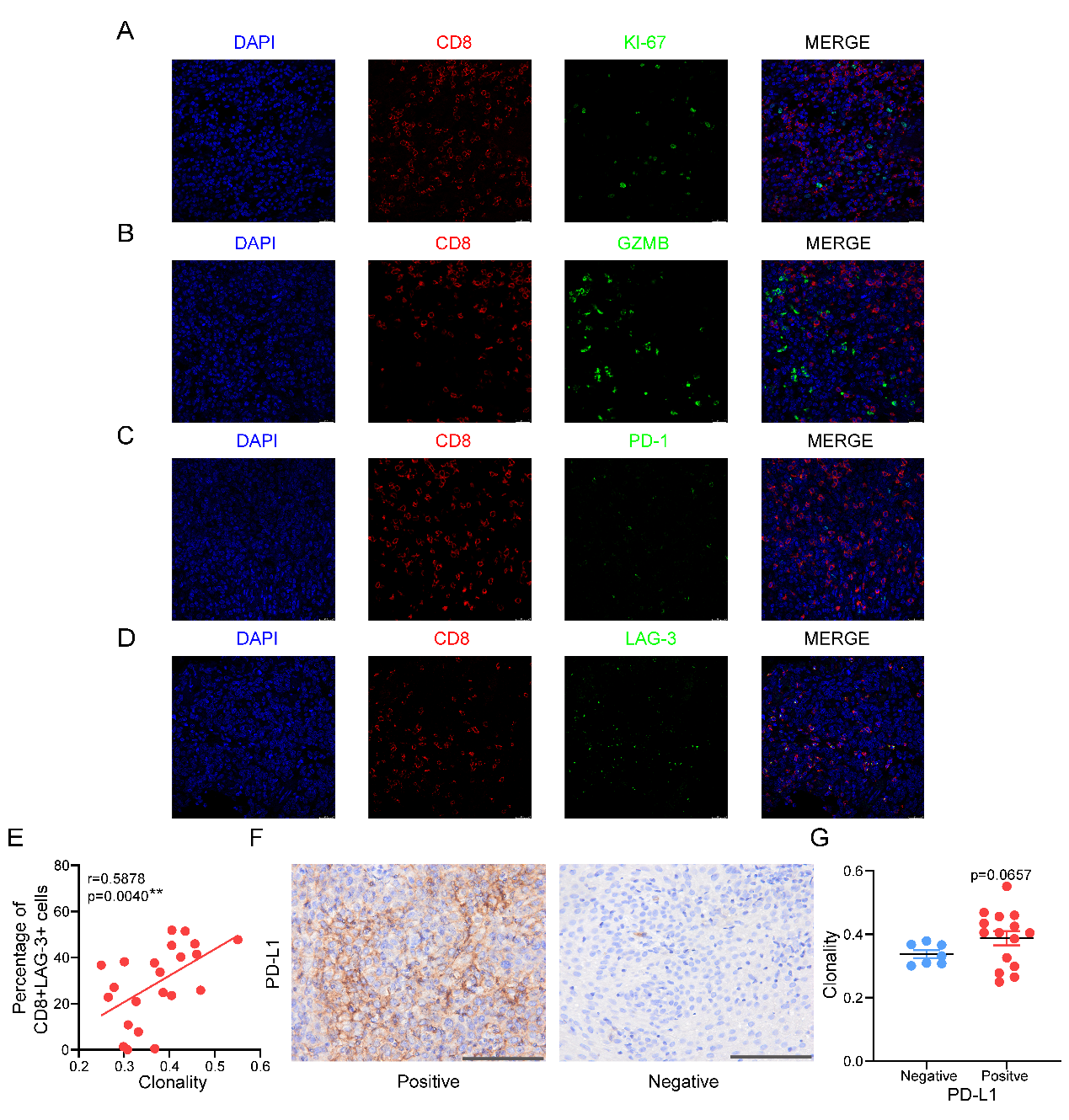

Supplement: Supplementary file 11 — Supplementary file11 (DOCX 1621 KB) [file 262_2023_3615_MOESM11_ESM.docx]
